# Age-related changes in peripheral T-cell subpopulations in elderly individuals: An observational study

**DOI:** 10.1515/biol-2022-0557

**Published:** 2023-02-09

**Authors:** Xiao-Qing Quan, Lei Ruan, Hai-Rong Zhou, Wei-Liang Gao, Qing Zhang, Cun-Tai Zhang

**Affiliations:** Department of Geriatrics, Department of General Practice, Shenzhen Longhua District Central Hospital, The Affiliated Central Hospital of Shenzhen Longhua District, Guangdong Medical University, Shenzhen, 518000, China; Department of Geriatrics, Tongji Hospital, Tongji Medical College, Huazhong University of Science and Technology, Wuhan, 430030, China; Department of Neurology, Tongji Hospital, Tongji Medical College, Huazhong University of Science and Technology, Wuhan, 430030, China

**Keywords:** T-cell subsets, immunosenescence, elderly, aging

## Abstract

The age-related decline in T-cell function among elderly individuals remains unclear. We thus investigated the interrelationship between T-cell subsets and age to identify the changes in T-cell phenotypes and develop an age prediction model for the elderly population. A total of 127 individuals aged >60 years were divided into three groups (youngest-old group, 61–70 years, *n* = 34; middle-old group, 71–80 years, *n* = 53; and oldest-old group, ≥ 81 years, *n* = 40). The percentage of CD8+CD28− cells (*P =* 0.001) was highest in the oldest-old group and then followed by the middle-old group, while the youngest-old group was the lowest. Both females and males demonstrated significant decreases in the absolute counts of CD4+CD45RA+ cells (*P =* 0.020; *P =* 0.002) and CD8+CD28+ cells (*P =* 0.015; *P =* 0.005) with age. Multivariate linear regression showed that the percentage of CD8+CD28− cells (*P* < 0.001) was an independent predictor of aging after adjusting for sex, body mass index, hospitalization duration, smoking, drinking, chronic medical illness, and medications at admission. In conclusion, our results suggest that aging in elderly individuals is accompanied by a decrease in the counts of T-cell subpopulations. CD8+CD28− cells may be potential targets for elderly individuals in antiaging-related immunosenescence.

## 1 Introduction

Aging is a varied and complex process that occurs with the decline in the immune system [1,2]. T-cell subsets play a key role in the evaluation of immune status and disease course [3]. The reference values of T-cell subpopulations are currently being widely utilized to evaluate the alteration in immune status. Immunosenescence is an inevitable process characterized by the deficiency of the T-cell response as individuals age, which has been a major hallmark of aging.

Aging-related changes in T-cell phenotypic compositions are well-accepted phenomena. Studies have demonstrated that older age is associated with an inversion of the CD4/CD8 ratio, a reduction in naive T cells, and an expansion in memory and effector T cells [2,4]. This process is accompanied by the reduced expression of membrane surface molecules, such as CD45RA, CD27, and CD28 [5,6]. The expression of CD45RA differentiation markers is usually associated with naive T cells [7]. It is recognized that CD45RA re-expressing memory cells display senescence-related proliferative defects but can effectively secrete homeostatic cytokines [8]. CD27 is an immune-checkpoint receptor and plays an important role in T-cell development, activation, and survival [9]. CD28 is involved in T-cell clonal expansion, metabolic activity, and IL-2 production [10]. The loss of CD28 expression is a key indicator of replicative senescence of human T cells [11]. The above molecules have been identified as the main phenotypes involved in T-cell immunosenescence and how they change with age merit further exploration.

Age-related changes in T-cell subsets could be utilized to predict the diagnosis and prognosis of diseases, including autoimmune diseases, cancers, pathogen infections, and primary immunodeficiencies [12,13,14,15]. Immunosenescence is the primary driver of age-related diseases, such as infections, cancers, and cardiovascular diseases [16,17,18]. Modifiable risk factors, such as nutritional status, physical activity, or dietary habits, can affect the senescence of the immune system [19,20]. The T-cell subset composition with aging plays a pivotal role in determining the successful and appropriate immune response of the host. To further explore the implication of senescent T-cell phenotypes in the aging process, we investigated the interrelationship between T-cell subpopulations and age and developed an age prediction model for the elderly population.

## 2 Materials and methods

### 2.1 Study design and participants

This retrospective study consecutively recruited 127 elderly inpatients aged >60 years at the Department of Geriatrics, Tongji Hospital, Tongji Medical College, Huazhong University of Science and Technology from December 2014 to February 2019. The patient population was separated into three age groups: 61–70 years, 71–80 years, and ≥81 years.

The exclusion criteria included the following: (1) serious infections, (2) medication regimens that could influence the immune system, (3) malignant tumor diseases, (4) autoimmune diseases, and (5) HIV infection. Patients who met any of the exclusion criteria were not enrolled in this study. Physical activity in the 7 days before admission was assessed using the physical activity scale for the elderly (PASE) scores [21]. The hospitalization duration refers to the number of days between admission and discharge.


**Informed consent:** Informed consent has been obtained from all individuals included in this study.
**Ethical approval:** The research related to human use has been complied with all the relevant national regulations, institutional policies, and in accordance with the tenets of the Helsinki Declaration and has been approved by the Ethics Committee of Tongji Hospital (ID: TJ-C20141112).

### 2.2 Flow cytometric analysis

Laboratory tests were completed by the Department of Laboratory Medicine, Tongji Hospital. Peripheral blood specimens were collected into vacutainer tubes containing EDTA as anticoagulant. Peripheral blood mononuclear cells were obtained by density centrifugation using Lymphoprep (StemCell Technologies, Vancouver, BC, Canada). Samples were stained with the antibody cocktail for 30 min at 4°C to analyze antigen expression: CD45 Pacific Orange/CD4 Pacific Blue/CD28 PE/CD45 RA FITC/CD25 PerCP-Cy5.5 (Thermo Fisher Scientific, Waltham, MA, USA), and CD3 APC-H7/CD8 APC (BD Biosciences, San Jose, CA, USA). The proportions of T-cell subsets were acquired from a BD LSRFortessa flow cytometer and analyzed using BD FACS Diva 6.2 software. Absolute T subpopulation numbers were calculated as the cell proportion multiplied by the absolute number of lymphocytes. The absolute counts of lymphocytes were detected by the automatic blood cell analyzer (XN-1000, Sysmex, Kobe, Japan). Treg cells were assessed as CD3+CD4+CD25+CD127^dim+^ events. Isotype controls and FMO controls were used as appropriate.

### 2.3 Data analysis

Categorical data were described as the frequency (percentage). Continuous variables are presented as medians with interquartile range (IQR) or means with standard deviation (SD) where appropriate. Comparisons among multiple groups were analyzed by Kruskal–Wallis *H* test, and between two groups were by Mann–Whitney *U* test. A chi-square test was used to analyze categorical data. Spearman rank correlation coefficient (*r*) and associated *P* values were calculated to determine the correlations between age and T-cell subpopulations. The unstandardized regression coefficient (*B*) was evaluated using linear regression to indicate the effect size of independent variables on dependent variables. For the decline and rise of the expression of T-cell markers with age, coefficient *B* represents the pace at which they change over time. Multiple linear regression was performed using a stepwise approach to develop the age prediction models. *P* values less than 0.05 were considered to be statistically significant. Statistical analyses were conducted by SPSS 24.0 (SPSS, University Chicago, IL, USA) and GraphPad Prism 8.0 (GraphPad Prism, San Diego, CA, USA).

## 3 Results

### 3.1 Profiles of samples

The baseline characteristics of the patients are shown in Table 1. The study sample consisted of 127 subjects with a mean age of 75.25 ± 9.34 years, 101 (79.5%) of whom were males. There were three age groups: youngest old, 61–70 years (*n* = 34, aged 64.00 ± 2.37); middle old, 71–80 years (*n* = 53, aged 73.74 ± 2.51); and oldest-old, ≥ 81 years (*n* = 40, aged 87.83 ± 4.22). Among the three groups, the oldest-old group had the lowest PASE scores (*P <* 0.001) and mean body mass index (BMI) (*P* = 0.003). Hospitalization duration was similar in the youngest-old group and the middle-old group but increased in the oldest-old group (*P <* 0.001). There was no significant difference in other demographic variables between groups.

**Table 1:** Demographic characteristics of study population

Variables	Youngest old (61–70 years; *n* = 34)	Middle old (71–80 years; *n* = 53)	Oldest old (≥81 years; *n* = 40)	*P*-value
Age (years)	64.00 ± 2.37	73.74 ± 2.51^a^	87.83 ± 4.22^a^	**<0.001**
Sex, male (%)	27 (79.4%)	42 (79.2%)	32 (80.0%)	0.996
PASE scores	176.08 (84.88–201.25)	140.20 (64.60–153.90)^a^	25.50 (17.07–66.58) ^a,b^	**<0.001**
BMI (kg/m^2^)	23.74 ± 2.63	23.03 ± 2.61	22.15 ± 2.31 ^a^	**0.003**
Hospitalization duration (days)	10.00 (4.75–14.25)	11.00 (5.00–17.00)	18.00 (12.25–24.50)^a,b^	**<0.001**
Smoking (%)	12 (35.3%)	16 (30.2%)	11 (27.5%)	0.765
Drinking (%)	9 (26.5%)	11 (20.8%)	5 (12.5%)	0.311
Chronic medical illness				
Hypertension (%)	13 (38.2%)	27 (50.9%)	25 (62.5%)^a^	0.115
Diabetes (%)	7 (20.6%)	14 (26.4%)	12 (30.0%)	0.652
Cardiovascular disease (%)	16 (47.1%)	26 (49.1%)	20 (50.0%)	0.968
Cerebrovascular disease (%)	9 (26.5%)	14 (26.4%)	11 (27.5%)	0.992
Chronic obstructive pulmonary disease (%)	6 (17.6%)	4 (7.5%)	9 (22.5%)^b^	0.118
Chronic liver disease (%)	3 (8.8%)	1 (1.9%)	2 (5.0%)	0.329
Chronic kidney disease (%)	4 (11.8%)	3 (5.7%)	7 (17.5%)	0.187
Severe infection (%)	0 (0.0%)	0 (0.0%)	0 (0.0%)	—
Medications at admission				
Antihypertensive drugs	13 (38.2%)	24 (45.3%)	22 (55.5%)	0.345
Hypoglycemic agents/insulin	7 (20.6%)	13 (24.5%)	11 (27.5%)	0.788
Statins	11 (26.8%)	18 (34.0%)	17 (42.5%)	0.601
Anti-thrombotic agents	13 (38.2%)	19 (35.8%)	20 (50.0%)	0.363
Oral antibiotics	1 (2.9%)	1 (1.9%)	1 (2.5%)	0.949

Data are presented as mean ± standard deviation, median (interquartile range), and frequency (percentage). *P*-value was calculated among the three groups and statistically significant *P*-values are marked in bold. BMI, body mass index; PASE, physical activity scale for the elderly.

^a^ *P* < 0.05 compared with the youngest-old group.

^b^ *P* < 0.05 compared with the middle-old group.

### 3.2 Dynamic changes in T-cell subsets in different age groups

**Figure A1:**
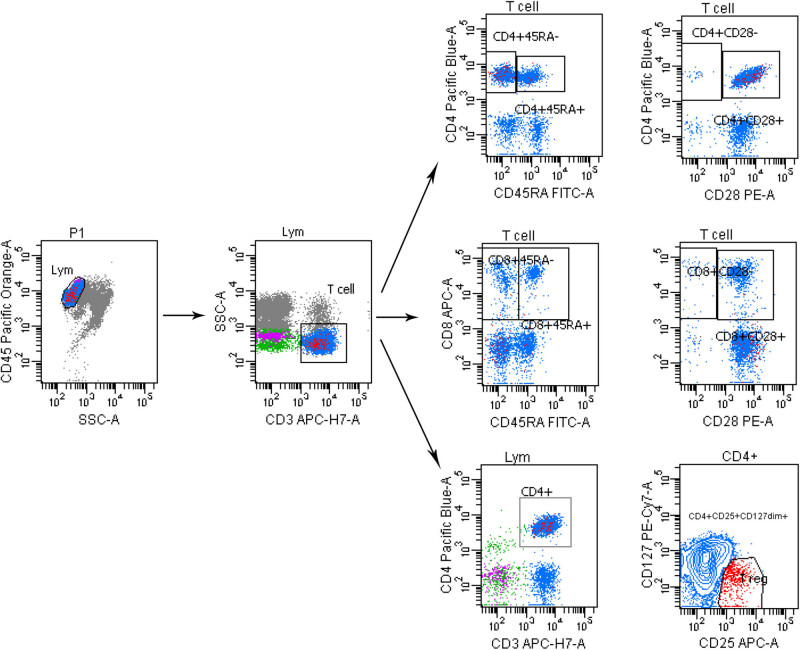
Gating strategies for flow cytometry.

Gating strategies for flow cytometry are shown in Figure A1. The absolute counts of lymphocytes (*P* = 0.021), T cells (*P* = 0.006), CD4+ cells (*P* = 0.005), CD4+CD25+CD127^dim+^ cells (*P* = 0.026), CD4+CD28+ cells (*P* = 0.001), CD4+CD45RA+ cells (*P* < 0.001), CD8+CD28+ cells (*P* = 0.007), and CD4/CD8 ratio (*P* = 0.037) were significantly different in the three groups, which were significantly lower in the oldest-old group than in the youngest-old group (*P* < 0.05) and the middle-old group (*P* < 0.05). Absolute counts in different age groups are shown in Table 2.

**Table 2:** Absolute counts of T-cell subsets in different age groups

T-cell subsets (/μl)	Youngest old (61–70 years; *n* = 34)	Middle old (71–80 years; *n* = 53)	Oldest old (≥81 years; *n* = 40)	*P*-value
Lymphocytes	1610.00 (1330.00–2015.00)	1700.00 (1225.00–2010.00)	1265.00 (935.00–1697.50) ^a,b^	**0.021**
T cells (CD3+CD45+)	1073.84 (852.36–1399.15)	1044.12 (744.35–1284.32)	717.04 (541.98–1145.76)^a,b^	**0.006**
CD4+ cells	606.45 (402.20–790.92)	525.41 (330.80–702.60)	394.33 (225.58–594.15)^a,b^	**0.005**
CD8+ cells	364.68 (305.35–580.17)	426.50 (250.70–587.42)	353.02 (203.69–493.26)	0.435
CD4+CD25+CD127^dim+^cells	68.59 (52.95–92.38)	65.17 (43.31–92.16)	46.94 (29.45–76.72)^a,b^	**0.026**
CD4+CD28+ cells	566.22 (350.45–706.50)	491.41 (299.49–661.30)	340.11 (185.80–524.67)^a,b^	**0.001**
CD4+CD28− cells	27.91 (7.56–52.03)	23.20 (10.48–55.78)	36.60 (15.64–56.83)	0.318
CD4+CD45RA+ cells	142.99 (80.18–256.00)	113.43 (49.78–207.20)	69.54 (36.65–125.28)^a,b^	**<0.001**
CD4+CD45RA− cells	412.05 (315.25–578.85)	382.55 (266.81–524.69)	289.56 (201.85–480.58)^a^	**0.011**
CD8+CD28+ cells	216.72 (148.36–310.98)	178.54 (144.36–289.30)	139.36 (90.93–210.65)^a,b^	**0.007**
CD8+CD28− cells	166.06 (97.00–244.66)	194.77 (73.15–289.67)	178.99 (102.83–270.51)	0.686
CD8+CD45RA+ cells	157.12 (81.41–213.36)	132.57 (100.07–197.54)	123.89 (56.44–188.67)	0.477
CD8+CD45RA− cells	214.26 (142.37–363.62)	256.81 (138.32–350.78)	199.18 (126.67–325.14)	0.584
CD4/CD8 ratio	1.44 (1.06–2.00)	1.31 (0.80–1.88)	0.97 (0.70–1.59)	**0.037**

Data are represented as median (interquartile range). *P*-value was calculated among the three groups and statistically significant *P*-values are marked in bold.

^a^ *P* < 0.05 compared with the youngest-old group.

^b^ *P* < 0.05 compared with the middle-old group.

As shown in Table 3, the percentages of CD8+ cells and CD8+CD28− cells in the oldest-old group were significantly increased (*P <* 0.05) compared with the youngest-old group, while the percentages of CD4+CD28+ cells and CD4+CD45RA+ cells, were decreased (*P <* 0.05). Moreover, the percentage of CD4+CD28− cells in the oldest-old group was significantly higher than those in the youngest-old group (*P* < 0.05) and the middle-old group (*P* < 0.05)

**Table 3:** Proportions of T-cell subsets in different age groups

Percentages of T-cell subsets (%)	Youngest old (61–70 years; *n* = 34)	Middle old (71–80 years; *n* = 53)	Oldest old (≥81 years; *n* = 40)	*P*-value
T cells (CD3+CD45+)/lymphocytes	68.40 (60.00–75.55)	65.50 (57.50–73.05)	62.90 (50.10–71.10)	0.176
CD4+ cells/T cells	57.75 (49.22–62.87)	53.80 (40.80–61.10)	49.55 (39.98–59.50)	0.111
CD8+ cells/T cells	35.80 (30.25–41.45)	41.50 (32.55–51.30)	48.30 (35.50–54.30)^a^	0.013
CD4+CD25+CD127^dim+^cells/T cells	6.50 (5.00–7.65)	7.00 (5.55–8.35)	6.55 (4.78–8.35)	0.659
CD4+CD28+ cells/T cells	53.40 (41.60–61.60)	48.60 (37.75–59.30)	44.30 (32.70–56.90)^a^	0.045
CD4+CD28− cells/T cells	2.30 (0.70–4.65)	2.60 (1.05–5.60)	5.10 (1.95–11.97)^a,b^	0.019
CD4+CD45RA+ cells/T cells	13.2 (8.00–20.65)	13.80 (6.55–17.65)	8.85 (3.15–16.98)^a^	0.030
CD4+CD45RA− cells/T cells	44.10 (32.95–47.75)	39.70 (31.15–44.3)	39.90 (32.13–49.45)	0.359
CD8+CD28+ cells/T cells	19.30 (14.15–26.45)	18.30 (14.75–23.70)	18.60 (13.90–25.18)	0.723
CD8+CD28− cells/T cells	13.80 (10.70–20.10)	19.80 (11.50–30.75)^a^	24.90 (19.10–34.45)^a^	0.001
CD8+CD45RA+ cells/T cells	14.3 (8.90–18.90)	13.1 (10.05–19.35)	18.20 (9.20–22.65)	0.291
CD8+CD45RA− cells/T cells	21.20 (15.60–28.60)	23.70 (17.1–29.85)	27.70 (20.55–35.95)	0.055

Data are represented as median (interquartile range). *P*-value was calculated among the three groups and statistically significant *P*-values are marked in bold.

^a^ *P* < 0.05 compared with the youngest-old group.

^b^ *P* < 0.05 compared with the middle-old group.

### 3.3 Correlations between age and T-cell subsets

**Figure 1:**
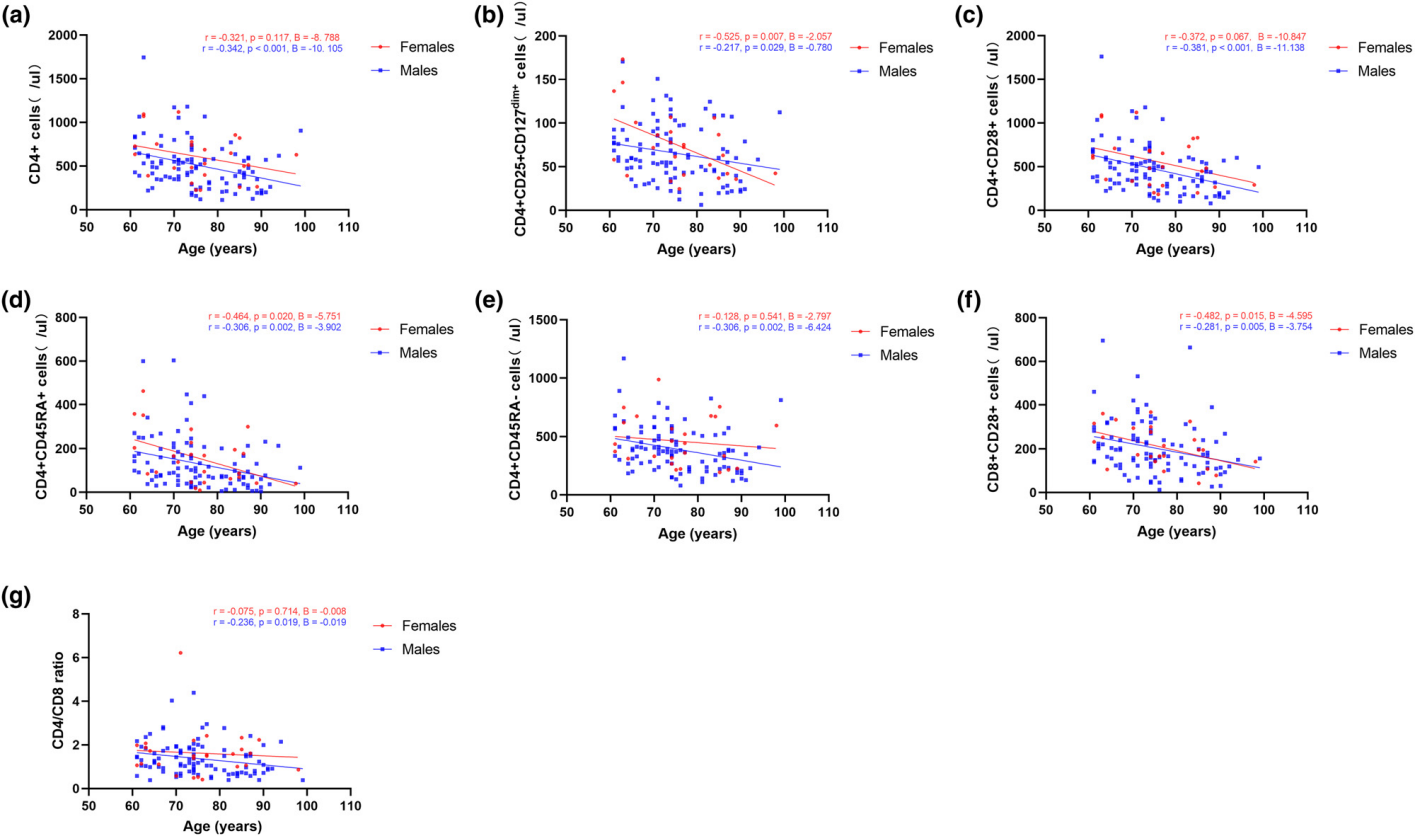
Correlation analysis between absolute counts of T-cell subsets and age. (a–g) Correlation between age and absolute counts of CD4+ cells, CD4+CD25+CD127^dim+^ cells, CD4+CD28+ cells, CD4+CD45RA+ cells, CD4+CD45RA− cells, CD8+CD28+ cells, and CD4/CD8 ratio. Red indicates females, and blue indicates males.

There is skewness in the female/male ratio, so we used different colors to represent different sexes in Spearman's correlation analysis. Both females and males demonstrated significant decreases in the absolute counts of CD4+CD25+CD127^dim+^ cells (*r =* −0.525, *P =* 0.007, *B* = −2.057; *r =* −0.217, *P =* 0.029, *B* = −0.780), CD4+CD45RA+ cells (*r =* −0.464, *P =* 0.020, *B* = −5.751; *r =* −0.306, *P =* 0.002, *B* = −3.902), and CD8+CD28+ cells (*r =* −0.482, *P =* 0.015, *B* = −4.595; *r =* −0.281, *P =* 0.005, *B* = −3.754) with age. Decreases in the absolute counts of CD4+ cells (*r =* −0.342, *P <* 0.001, *B* = −10.105), CD4+CD28+ cells (*r =* −0.381, *P <* 0.001, *B* = −11.138), CD4+CD45RA− cells (*r =* −0.306, *P =* 0.002, *B* = −6.424), and CD4/CD8 ratio (*r =* −0.236, *P =* 0.019, *B* = −0.019) were observed with increasing age only in the male elderly. Correlation analysis between absolute counts of T-cell subsets and age is shown in Figure 1.

**Figure 2:**
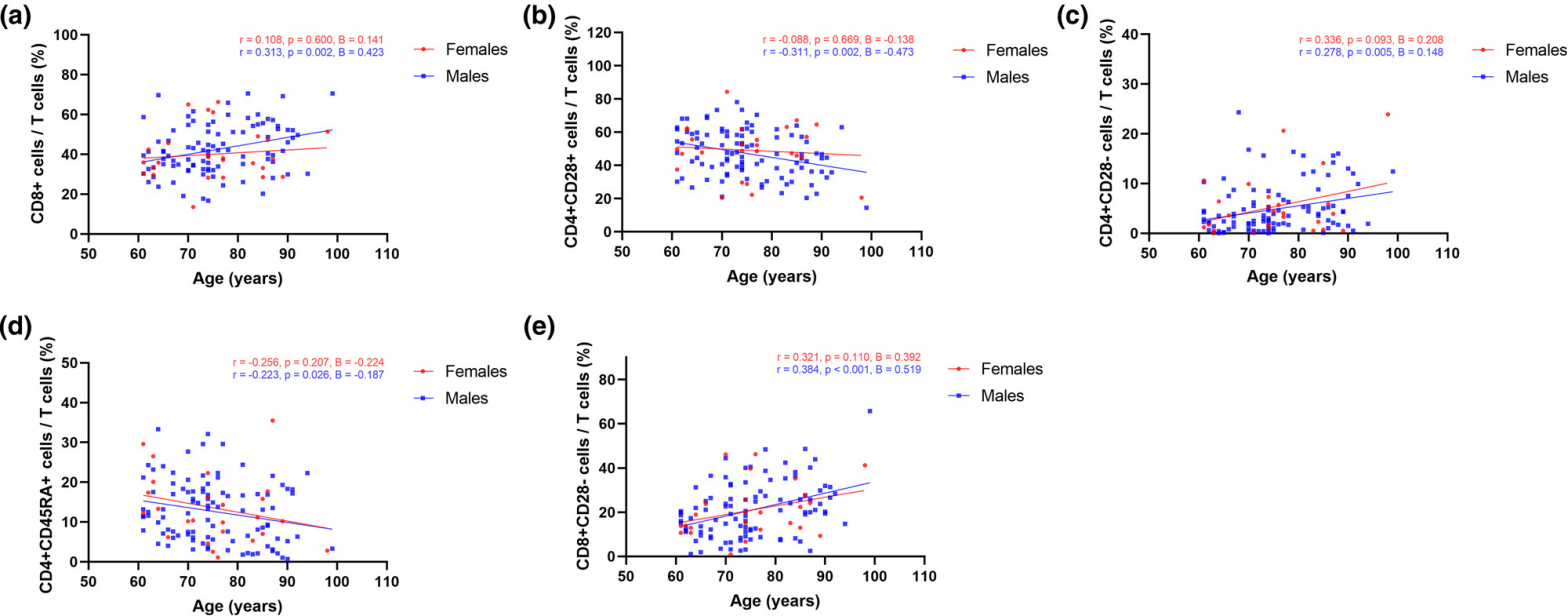
Correlation analysis between proportions of T-cell subsets and age. (a–e) Correlation between age and proportions of CD8+ cells, CD4+CD28+ cells, CD4+CD28− cells, CD4+CD45RA+ cells, and CD8+CD28− cells. Red indicates females, and blue indicates males.

For T-cell proportions, no independent correlation between age and T-cell subsets was shown in females. The percentages of CD8+ cells (*r =* 0.313, *P =* 0.002, *B* = 0.423), CD4+CD28− cells (*r =* 0.278, *P =* 0.005, *B* = 0.148), and CD8+CD28− cells (*r =* 0.384, *P <* 0.001, *B* = 0.519) were all positively correlated with aging in males. In contrast, a decreasing trend in the percentages of CD4+CD28+ cells (*r =* −0.311, *P =* 0.002, *B* = −0.473) and CD4+45RA+ cells (*r =* −0.223, *P =* 0.026, *B* = −0.187) was found to be associated with aging in males. Correlation analysis between proportions of T-cell subsets and age is shown in Figure 2.

### 3.4 Multiple linear regression analysis

Table 4 features the multivariate linear regression showing independent predictor variables for aging, including PASE scores (*B* = −0.06, *P* < 0.001), history of hypertension (*B* = 3.37, *P* = 0.012), the percentages of total T cells (*B* = −0.12, *P* = 0.018), and CD8+CD28− cells (*B* = 0.24, *P* < 0.001). Further adjusted for sex, BMI, hospitalization duration, smoking, drinking, chronic medical illness, and medications at admission, PASE scores (*B* = −0.07, *P* < 0.001), history of hypertension (*B* = 6.51, *P* = 0.024), and the percentage of CD8+CD28− cells (*B* = 0.20, *P* < 0.001) were still independent predictors.

**Table 4:** Association between T-cell subsets, clinical data, and age using multivariable linear regression analysis

Biomarker	Model 1		Model 2	
	*B* (95% CI)	*P*-value	*B* (95% CI)	*P*-value
PASE scores	−0.06 (−0.08 to –0.03)	**<0.001**	−0.07 (−0.09 to –0.04)	**<0.001**
Hypertension	3.37 (0.77 to 5.97)	**0.012**	6.51 (0.86 to 12.16)	**0.024**
T cells (CD3+CD45+)/lymphocytes (%)	−0.12 (−0.22 to–0.02)	**0.018**	−0.11 (−0.21 to 0.01)	0.052
CD8+CD28− cells/T cells (%)	0.24 (0.13 to 0.36)	**<0.001**	0.20 (0.08 to 0.32)	**<0.001**

Model 1: results of stepwise multiple linear regression; model 2: further adjusted for sex, BMI, hospitalization duration, smoking, drinking, chronic medical illness, and medications at admission. Statistically significant *P*-values are marked in bold. BMI, body mass index. PASE, physical activity scale for the elderly.

## 4 Discussion

By 2050, the world population aged 60 years and older will account for approximately 22%, more than double the 10% of the elderly population in 2010 [22]. As the aging population rapidly grows, the complex physiological process of aging leads to a serious increase in the social medical burden [22,23]. In this study, we measured T-cell subpopulations to identify aging-associated factors in elderly individuals and found that the percentage of CD8+CD28− cells was an independent predictor.

In accordance with previous studies, our results demonstrated that indicated alterations in CD4+ T-cell subsets with aging characterized by a decline in the expression of CD28 and CD45RA [24]. No significant differences in the absolute counts of T-cell subsets were observed between the youngest-old group and the middle-old group, which demonstrated a significant T-cell exhaustion in the elderly patients over 80 years old. However, the differences between the three age groups cannot determine when changes in T-cell markers start and further studies are necessary. Although our results showed no statistically significant correlation between the majority of T-cell subsets and age in the female elderly, similar trends were apparent in the male elderly. Our multivariate regression analysis was performed to adjust for the skewness in the data.

There was no significant difference in the expression of CD45RA on CD8+ T cells between the three age groups in this study. These findings indicated that the proliferation ability of CD4+ cells might be impaired more significantly with age than that of CD8+ cells in the elderly population [25]. It has been proposed that T cells can be divided into naive T cells (CCR7+CD45RA+), central memory T cells (CCR7+CD45RA−), effector memory T cells (CCR7−CD45RA−), and terminally differentiated T cells (CCR7−CD45RA+). The expression of CCR7 was not measured in our samples, which requires investigation in the future.

With increasing age, healthy individuals usually experience a loss of CD28 expression [26]. Similar to previous results [26,27], the percentages of CD8+CD28− cells and CD4+CD28− cells were highest in the oldest-old group and then followed by the middle-old group, while the youngest-old group was lowest. Lack of CD28 expression could inhibit T-cell survival and cytokine production in elderly individuals [11,28]. In addition, the results of multiple linear regression showed that CD8+CD28− was an independent index for predicting age. Regarding the correlations between age and proportions of T-cell subsets, the highest coefficient *B* for the percentages of CD8+CD28− cells indicates that this subpopulation has increased with aging at the fastest pace (Figure 2e). To date, CD8+CD28− T-cell alterations have been reported to be a strong contributor to age-related diseases such as diabetes [29], cardiovascular diseases [30], and chronic infections [31].

This present study indicated that the absolute count of CD4+CD25+CD127^dim+^ cells in the oldest-old group was significantly lower than that in the youngest-old group and the middle-old group, which was similar to previous reports [32]. Autoimmune diseases and inflammatory disorders such as rheumatoid arthritis [33], systemic lupus erythematosus [34], and coronavirus disease 2019 (COVID-19) infection [35] can also decrease CD4+CD25+CD127^dim+^ cells. The gradually decreased count of CD4+CD25+CD127^dim+^ cells might exacerbate dysregulation of immune homeostasis and impact the progression of diseases [36,37]. Further investigation into the role of CD4+CD25+CD127^dim+^ cells in elderly individuals is warranted.

Alterations in T-cell subsets with aging may lead to the development of chronic diseases and frailty status [38,39], and several lifestyle modifications can prevent or slow down the age-related decline in health [40]. Previous studies demonstrated that exercise induced increased expression of CD28 and CD45RA on the T-cell surface [41,42]. Vitamin supplementation could affect the levels of CD28+ T-cell subsets [43,44]. Caloric restriction has been shown to attenuate the age-related shift from naive to memory T cells in aged animals [45]. Regular physical exercise and appropriate nutritional interventions could help older populations avoid immune-related diseases, which merits further investigation.

The present study focused on the alteration of T-cell subsets with age in older people. There are some limitations to this study. First, the ratio of women to men in the included individuals stood at 1:4. Second, our analysis was based on a single-center retrospective study. Third, due to the retrospective design, CCR7 and cytokines cannot be complemented for detection and need further exploration. Finally, the major limitation of this study is the inability to provide isotype controls and FMO controls.

## 5 Conclusions

The present study suggests that aging in the elderly population is accompanied by a decrease in the count of T-cell subsets, characterized by a decline in the expression of CD28 and CD45RA. Furthermore, CD8+CD28− cells could be a useful factor in the age prediction model. This study reports the correlation between T-cell subpopulations and age among elderly individuals, which could be used to guide immunological evaluation for anti-aging strategies.
